# Enhancing crop resilience by harnessing the synergistic effects of biostimulants against abiotic stress

**DOI:** 10.3389/fpls.2023.1276117

**Published:** 2023-12-18

**Authors:** Anam Asif, Maratab Ali, Muslim Qadir, Rajmohan Karthikeyan, Zora Singh, Ravjit Khangura, Francesco Di Gioia, Zienab F. R. Ahmed

**Affiliations:** ^1^Integrative Agriculture, College of Agriculture and Veterinary Medicine, United Arab Emirates University, Abu Dhabi, United Arab Emirates; ^2^College of Agricultural Engineering and Food Science, Shandong University of Technology, Zibo, Shandong, China; ^3^School of Food and Agricultural Sciences, University of Management and Technology, Lahore, Punjab, Pakistan; ^4^Department of Plant Breeding and Genetics, Faculty of Agriculture, Lasbela University of Agriculture Water and Marine Sciences, Lasbela, Balochistan, Pakistan; ^5^Horticulture, School of Science, Edith Cowan University, Joondalup, WA, Australia; ^6^Department of Primary Industries and Regional Development, Government of Western Australia, Kensington, WA, Australia; ^7^Department of Plant Science, College of Agricultural Sciences, The Pennsylvania State University, College State, PA, United States

**Keywords:** bio-based compounds, abiotic stress, plants, microalgae, GABA

## Abstract

Plants experience constant exposed to diverse abiotic stresses throughout their growth and development stages. Given the burgeoning world population, abiotic stresses pose significant challenges to food and nutritional security. These stresses are complex and influenced by both genetic networks and environmental factors, often resulting in significant crop losses, which can reach as high as fifty percent. To mitigate the effects of abiotic stresses on crops, various strategies rooted in crop improvement and genomics are being explored. In particular, the utilization of biostimulants, including bio-based compounds derived from plants and beneficial microbes, has garnered considerable attention. Biostimulants offer the potential to reduce reliance on artificial chemical agents while enhancing nutritional efficiency and promoting plant growth under abiotic stress condition. Commonly used biostimulants, which are friendly to ecology and human health, encompass inorganic substances (e.g., zinc oxide and silicon) and natural substances (e.g., seaweed extracts, humic substances, chitosan, exudates, and microbes). Notably, prioritizing environmentally friendly biostimulants is crucial to prevent issues such as soil degradation, air and water pollution. In recent years, several studies have explored the biological role of biostimulants in plant production, focusing particularly on their mechanisms of effectiveness in horticulture. In this context, we conducted a comprehensive review of the existing scientific literature to analyze the current status and future research directions concerning the use of various biostimulants, such as plant-based zinc oxide, silicon, selenium and aminobutyric acid, seaweed extracts, humic acids, and chitosan for enhancing abiotic stress tolerance in crop plants. Furthermore, we correlated the molecular modifications induced by these biostimulants with different physiological pathways and assessed their impact on plant performance in response to abiotic stresses, which can provide valuable insights.

## 1 Introduction

Plants being sessile are constantly susceptible to various biotic and abiotic stresses, which adversely affect their development and yield performance (He et al., 2018; López-Valdez et al., 2022). Plants experience constant exposure to a wide range of biotic (bacteria, fungi, nematodes, herbivores, and weeds) and abiotic stresses (salinity, waterlogging, drought, high temperature, and ultraviolet B radiation) (Cirillo et al., 2022). They respond to these stresses using adaptive mechanisms that involve multifarious and interlinked cross-talks for survival. Notably, such as strsses could potentially impact various physiological, biochemical, and molecular mechanisms in the plant life cycle, ranging from seed germination to flowering, resulting in heavy yield losses (Pareek et al., 2010; Ranjan et al., 2021). Abiotic stresses can adversely impact the overall yield of several crop plants (Pareek et al., 2010; Kar and Raichaudhuri, 2021). Current climate change and global warming are obfuscating the food chain by existing negative influences on crop yield *via* increased biotic and abiotic stresses (Yousaf et al., 2022). Increasing global population predicted to reach 9.7 billion by 2050 according to the United Nations Department of Economic and Social Affairs, is resulting in a soaring demand for food (UNDESA, 2019). In this scenario, agricultural production needs to be increased by 60%–70%, and crop losses due to abiotic and biotic stresses need to be significantly reduced (Moenne and González, 2021). Extensive utilization of biostimulant applications against various abiotic stresses such as drought, heat, salinity, and waterlogging, can enhance plant growth at several developmental stages.

**Figure 1:**
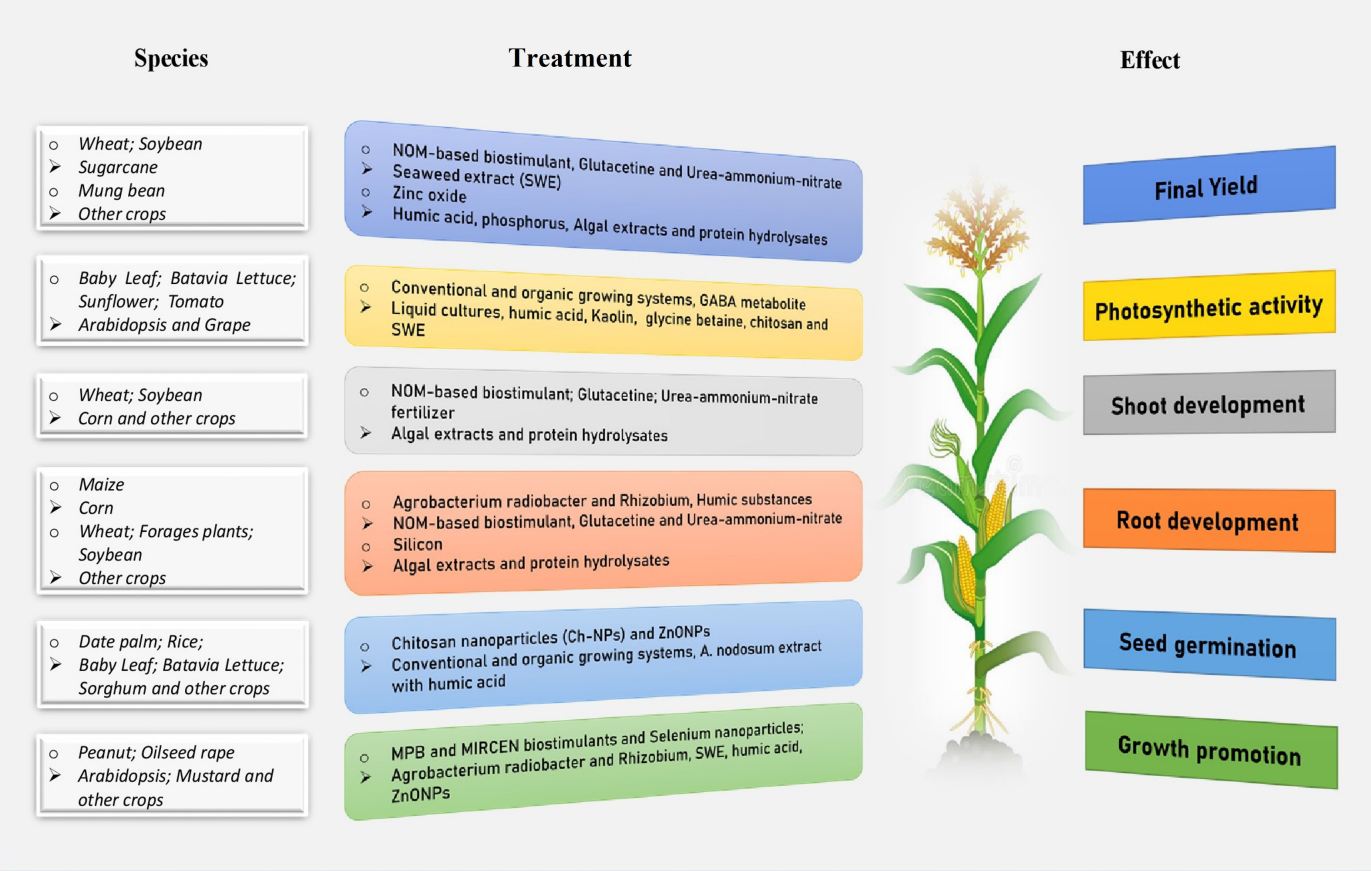
An integrated beneficial influence of biostimulants treatment on plants in different developmental stages and species under abiotic stress conditions.

Bio-based compounds including zinc oxide (Khan et al., 2018b; Azmat et al., 2022), silicon (Grusak et al., 2016; Araújo et al., 2022), silica (Ismail et al., 2022), selenium (Zhai et al., 2017; El-Badri et al., 2022), and γ-aminobutyric acid (GABA) (Nayyar et al., 2014; Balfagón et al., 2022), and melatonin (Pawar and Laware, 2018; Rajora et al., 2022) have diverse effects on plant growth and yield (**Figure 1**). Beyond their stress-counteracting properties, they can also confer biotic and abiotic stress tolerance to crop plants, thereby opening up a new horizon to support sustainable crop production in several agro-ecological areas of the world (Katarzyna, 2015).

The use of synthetic chemicals against biotic or abiotic stresses is likely to reduce soil fertility and above/below-ground biodiversity. Furthermore, the indiscriminate use of chemical stimulants and pesticides poses a severe risk to ecosystems and human health. It is important to identify the environmentally friendly compounds that might influence crop yield indirectly by triggering the defense system against biotic and abiotic stresses (Hidangmayum et al., 2019; Moenne and González, 2021). Therefore, employing environmentally safe methods is essential to reduce the antagonistic consequences of the agro-chemicals (Patil and Solanki, 2016; Poveda, 2021). For instance, plant-derived biostimulants can have a significant role in improving sustainable crop production (Zulfiqar et al., 2019). Such biostimulants can promote nutrient uptake and enhance plant growth to overcome different abiotic stresses by promoting bioactive compounds contents and antioxidant capacity of the several crops (Zulfiqar et al., 2020; Aldhanhani et al., 2022a; Aldhanhani et al., 2022b). The advantages of plant-based biostimulants undoubtedly open up new avenues for sustainable agriculture.

Numerous biostimulants have been developed and currently marketed, especially in the agricultural sector. Plant root system and leaf spray of three different tree species (*Quercus rubra*, *Betula pendula*, and *Fagus sylvatica*), for example, respond favorably to multiple biostimulant products marketed under the trade names Ģeneŕate, Çrop Set, Fulcrum, and Redicrop 2000 (De Vasconcelos and Chaves, 2019). Applying the biostimulant Stimulate® (contains 0.009% cytokinin, 0.005% gibberellin, and 0.005% auxin) on sugarcane stalks yield increased production and profitability index. Biostimulants such as Çarbonsolo® (contains 25% fulvic acids, 50% humic acids, 20% amino acids, and 2% water-soluble nitrogen), Retrosal® (contains calcium, zinc, and specific active ingredients), Terra-Sorb® (contains an amino acid product obtained by Enzymatic Hydrolysis), and Ķymon Plus®, which contain amino acids (e.g., arginine, serine, phenylalanine, alanine, aspartic acid, glycine, proline, hydroxyproline, glutamic acid, tryptophan, and valine) were used separately or in multiple combinations to treat maize crop, soybeans, lettuce and ryegrass via leaves. Application of these biostimulants on plants growing under water deficit, resulted in increased dry mass and leaf area (De Vasconcelos and Chaves, 2019).

In this review, we analyzed the current status on the use of various plant biostimulants, such as humic acids (Has) hydrolysates, seaweed extracts, chitosan, bacteria, zinc oxide, silicon, selenium, and GABA, in reducing the negative effects of various abiotic stresses on plant growth and yield of significant crop plants.

## 2 Role of bio-based compounds against biotic and abiotic stresses in plants

### 2.1 Zinc oxide

Zinc (Zn) is a crucial micronutrient, significantly contributes to plant growth, development, and yield, participating in several plant functions including protein synthesis. It can counteract the antagonistic effects of high temperature, heavy metal, and salt stresses on plants (Kambe et al., 2015; Ahmad et al., 2020). Additionally, zinc acts as a catalyst in the activity of various enzymes including DNA and RNA polymerases, dehydrogenases, transphosphorylases, and proteinases, given its involvement in the maintenance of membranous structure and cell division and chlorophyll production in plants (Vaghar et al., 2020).

Zinc oxide, by upregulating antioxidants and osmoprotectants, can help mitigate the negative effects of heat stress on mung bean crops. This, in turn, may lead to increased agricultural output and productivity, even under adverse environmental conditions (Kareem et al., 2022). Drought is an abiotic factor that significantly affects the grain yield in bread wheat crops (Eftekhari et al., 2017). Plants respond to drought conditions by exhibiting adaptive mechanisms at the morphological and physiological levels, enabling their survival and resilience to water deficits (Hayatu et al., 2014; Merwad et al., 2018). According to White and Broadley (2009), the application of zinc fertilizer treatment resulted in increased grain production and enhanced grain zinc level in cereal crops, addressing both yield and nutritional requirements. Zou et al. (2012) have determined the required nutritional zinc level. Plants’ zinc intake may influence their susceptibility to drought stress (**Table 1**).

**Table 1:** Effect of biostimulants in alleviating various abiotic stresses on different plant crops.

Species	Biostimulants	Stress	Beneficial effect	References
**Arabidopsis (*Arabidopsis thaliana*)**	Solid medium, liquid cultures, Seaweed extracts and humic acid	Drought, salinity or cold stress and heat stress	Early growth cell death, chloroplast degradation and activate Heat-Shock Proteins	Cha et al., 2020; Benito et al., 2022; Nephali et al., 2020
**Corn** **(*Zea mays*)**	NOM-based biostimulant	Drought and salt stress	Root, shoot weights and final yields	Sleighter et al., 2023
**(*Catharanthus roseus*)**	Chitosan nanoparticles (Ch-NPs)	Salinity stress	higher alkaloid accumulation, antioxidant capacity	Hassan et al., 2021
**Forages plants (Urochloa brizantha cv. Marandu and Megathyrsus maximum cv. Massai)**	Silicon	Water deficit	Nutrient efficiency, root	Araújo et al., 2022
**Grapevine** **(*Vitis vinifera*)**	Kaolin, glycine betaine, chitosan, seaweed	heat stress	photosynthetic activity, antioxidant capacity	Monteiro et al., 2022
**Baby Leaf lettuce and Batavia Lettuce (*Lactuca sativa)***	Bacillus subtilis PTB185, B. pumilus PTB180 and wollastonite	Drought stress	Germination rates and Chlorophyll fluorescence	Clément et al., 2023
**Lettuce**	protein hydrolysates	Salt and drought	Nitrogen metabolism and osmolytes	Nephali et al., 2020
**Maize**	Humic substances, HA, seed soaking	Salt and drought stress	Cell elongation in roots, antioxidants development	Hasanuzzaman et al., 2022; Nephali et al., 2020
**Mango** **(*Mangifera indica*)**	KCl, K2SO4 and biostimulants	Water deficit and nutrient deficiency	branch maturation	Araújo et al., 2022
**Mung bean** **(*Vigna radiata*)**	Zinc oxide	Heat stress	Final yield	Eftekhari et al., 2017
**Mustard** **(*Brassica nigra*)**	Foliar application of ZnONPs	Salt, drought stress, cadmium	Growth and antioxidants activities	Adrees et al., 2021; Priyanka and Venkatachalam, 2016
**Orange** **(*Citrus sinensis*)**	A. nodosum seaweed extract	Drought stress	Weight, quality and maturity	Battacharyya et al., 2015
**Olive** ***(Olea europaea*)**	Seaweed, glycine betaine and kaolin	Heat and drought	Vegetative and leaf nutraceutical trait	Graziani et al., 2022
**Oilseed rape** **(*Brassica napus*)**	Application of Selenium nanoparticles	Arsenic stress	Growth and development	El-Badri et al., 2022; Hu et al., 2022
**Peanut** **(*Arachis hypogaea*)**	Biostimulants; MPB and MIRCEN (*Bradyrhizobium*)	Drought, oxidative stress	ROS (reactive oxygen species); plant growth	Furlan et al., 2019
**Rice** ***(Oryza sativa)***	Foliar application of ZnONPs	Water deficit	Seed priming, scavenging ROS	Ghani et al., 2022; Mazhar et al., 2022
**Sunflower** **(*Helianthus annuus*)**	Application of GABA metabolite	Drought stress, heat/chilling	Chlorophyll and sugar level development	Ali et al., 2020; Abdel Razik et al., 2021
**Sorghum** **(*Sorghum bicolor*)**	Humic acid application	Salinity stress	Early seedlings	Ali et al., 2020; Abdel Razik et al., 2021
**Sugarcane** **(*Saccharum officinarum*)**	Seaweed extract (SWE)	Drought stress	stalk and final yield	Jacomassi et al., 2022
**Soybean** **(*Glycine max*)**	NOM-based biostimulant	Drought and salt stress	Root, shoot weights and final yields	Sleighter et al., 2023
**Tomato** **(*Solanum lycopersicum*)**	Soil and foliar applied biostimulants (NPK)	Salt and drought stress	Pollen viability and photosynthetic protectants	Koleška et al., 2017; Francesca et al., 2022
**Wheat** **(*Triticum aestivum)***	NOM-based biostimulant, Glutacetine;	Drought and salt stress	Root, shoot weights and spike	Liu et al., 2021; Sleighter et al., 2023
**Other crops**	*Agrobacterium radiobacter, Streptomyces, B. subtilis and Rhizobium*	Drought stress	Biomass accumulation, root and growth	Hamid et al., 2021
	Nutrient solution (hydroponics)	Salinity and temperature	Phenotypic changes in different stages	Drobek et al., 2019
	Biostimulants, humic acid extract	Drought and cold stress	stalk length and leaf color	Deolu-Ajayi et al., 2022; Franzoni et al., 2022
	Fungi seaweeds, Zn, Gas and BRs	Salinity and Waterlogging	Seed germination and flowering	Diaz-Vivancos et al., 2013; Cirillo et al., 2022
	Humic acid, phosphorus, algal extracts and protein hydrolysates	Water deficit, salt and oxidative stress	Roots and shoots development, increase crop yield and osmotic stress	Zhu, 2001; Çimrin et al., 2010; Van Oosten et al., 2017

In rice, seed priming with zinc oxide nanoparticles (ZnONPs) was found to mitigate water deficit, by altering antioxidant enzymes and osmolytes production (Mazhar et al., 2022). The foliar application of ZnONPs mitigates water stress in cucumber seedlings by altering the antioxidant mechanism, scavenging reactive oxygen species (ROS), and synthesizing osmolytes, resulting in overall improved crop growth (Ghani et al., 2022).

Owing to their eco-friendliness, ZnONPs have gained global recognition and can significantly promote cotton (*Gossypium hirsutum*) growth, physiological indexes, and enzyme activities with decreased malondialdehyde (MDA) levels in plants (Priyanka and Venkatachalam, 2016; Feng et al., 2019; Adrees et al., 2021). ZnONPs applied to mustard plants can promote growth and antioxidant enzyme activities (Rao and Shekhawat, 2014). Foliar exposure of ZnONPs increased the growth and stress tolerance mechanism of wheat plant demonstrated by increased antioxidant enzyme activity and oxidative stress markers in the leaves under water deficit (Adrees et al., 2021; Zulfiqar and Ashraf, 2021a). Additionally, arsenic, cadmium, and salt stresses were reduced upon ZnONPs application to wheat and rice, which altered crop redox status, antioxidant, and morphophysiological mechanisms (Adrees et al., 2021; Faizan et al., 2021) as presented in **Table 1**.

Genetic engineering, and molecular marker-assisted selection, specifically quantitative trait locus mapping, represent long-term approaches to reducing the negative effects of abiotic stress on rice crops (Faizan et al., 2021). Nanoparticles (NPs) utilization in agriculture can reduce the overreliance on chemical fertilizers and significantly enhance seed germination and plant growth in different crops (Acharya et al., 2019). Overall, future studies should explore ZnONPs utilization for increasing the stress tolerance and production of agricultural plants, especially in areas with drought and salt issues, which have become major problems in most cultivated lands in dry climatic regions. While incorporating biotechnologies in these studies to investigate the molecular mechanism underlying plant defensive systems is crucial, applying NP mediation targeting biomolecules to obtain novel cultivars tolerant to varying ecological challenges is also necessary.

### 2.2 Silicon

Silicon (Si) is a micronutrient, can increase plant tolerance to abiotic stresses (e.g., higher temperature, UV radiation, metal toxicity, nutrient deficiency and water deficit) via its potential to synthesize phytoalexins and phenolics in response to these stresses (Tripathi et al., 2020; Awasthi et al., 2022; Christian et al., 2022). Silicon is the second most abundant element in the soil after oxygen, comprising 60 to 70% of the soil mass in the form of SiO_2_, and it contributes significantly to plant nutrient uptake, nutrient remobilization, and protection against biotic and abiotic stresses (Richmond and Sussman, 2003; Christian et al., 2022).

Silicon reduces the negative effects and nutritional shortages on forage (*Urochloa brizantha* and *Megathyrsus maximum)* crops. The emerging climate change is responsible for the prevailing nutritional deficiency related to nitrogen, phosphorus, and calcium in many forage growing parts (Katz et al., 2021). The benefits of silicon in pasturelands may be amplified using fertigation to attenuate water deficit, which is a common condition in drought periods in several regions worldwide that results in low forage production, regardless of crop species (Buchelt et al., 2020; Araújo et al., 2022) (**Table 1**). As determined by liquid chromatography mass spectrometry-based metabolomics, salinity tolerance in silicon treated tomato plants was attributed to the induced accumulation of primary metabolites in treated plants, which acted as osmotic and photosynthetic protectants (Le et al., 2014; Chele et al., 2021). Likewise, Christian et al. (2022) demonstrated the positive role of silicon in improving plant nutrition and tolerance against drought stress, leading to improved wheat grain yield and overall crop quality.

Application of silicon on various crops increased tolerance against salinity and drought stress by modulating the metabolic mechanisms (Wang et al., 2021). Notably, silicon NPs (SiNPs) can effectively alleviate the antagonistic effects of abiotic stresses, resulting in reduced postharvest losses in climate resilient crops by suppressing oxidative injury (Le et al., 2014; Yousaf et al., 2022). The positive effects of SiNPs were also correlated with improved resistance mechanisms in salt-stressed mango trees (Elsheery et al., 2020).

Silicon plays a critical role in salt stress in cucumber plants by improving tolerance and nutrients uptake, resulting in increased mechanical support to plant shoots and leaf edges (Khan et al., 2018a; Zhu et al., 2019). The foliar application of silicon has several other beneficial effects on plants growing under salt conditions, including improved ion balance, reduced membrane injury, osmotic concentrations, enhanced the production of antioxidant enzymes to degrade oxidative chemicals, better morphological traits, and enhanced growth of liquor ice (*Glycyrrhiza glabra* L.) by reducing plant sodium ion uptake (Sapre and Vakharia, 2016; Shen et al., 2022). Silicon accelerates the activity of enzymes such as peroxidase, polyphenol oxidase, phenylalanine ammonia-lyase, and acyltransferase related to lignin biosynthesis. Silicon application enhances lignification in rice crop (Fleck et al., 2011; Dhiman et al., 2021). Adequate seed germination is critical for crop establishment. Moisture availability, temperature, nutrient availability, and seed quality can influence seed germination rate (Hassan et al., 2023). Silicon application as a priming agent enhanced the seed germination rate by up to 24% under drought conditions (Shi et al., 2016; Ayed et al., 2022). Silicon emerges as a potential solution for alleviation ecological stresses and mitigating soil nutrient depletion, making it a viable option for promoting sustainable agriculture. Comprehensive molecular and genetic research is necessary to provide theoretical support for silicon supplementation in crop cultivation. This research can help elucidate the mechanisms by which silicon mediates the reduction of nutritional imbalance stress in plants. To maximize the effectiveness of silicon in the agriculture sector, future studies should establish ideal silicon nutrition timing and dosages for certain crops cultivated in various edaphic and climatic conditions.

### 2.3 Selenium

Selenium (Se) is a valuable trace element that positively affects crop health and stress tolerance when applied at low concentrations (Awasthi et al., 2022). Several researchers have demonstrated its defensive role against various abiotic stresses in higher plants. The application of selenium NPs (SeNPs) favors the remediation of suppressed rapeseed growth and development under abiotic stress (El-Badri et al., 2022). The supplementation of organic selenium significantly increased resistance to arsenic stress/toxicity in radish (*Raphanus sativus*) by enhancing superoxide dismutase and peroxidase activities and soluble protein, chlorophyll, and proline levels while decreasing MDA levels (Hu et al., 2022). Selenium supplementation can also limit metal translocation to roots and shoots, thus promoting tolerance against metal stress. Furthermore, Hasanuzzaman et al. (2022) highlighted the alleviating effect of selenium on metal/metalloid toxicity in plants via improved tolerance involving various mechanisms. Selenium activates hormones related to biosynthesis to modulate the root structure, enabling suppressed metal uptake. Selenium supplementation also improves photosynthesis under metal/metalloid stress via inhibited pigment degradation, enhanced antioxidant defense related enzymatic activities, better stomatal function, and photosystem activation (Feng et al., 2020; Hasanuzzaman et al., 2022).

SeNPs significantly contribute to the activation of plant defense system in response to several stress factors (El-Saadony et al., 2021). Several researchers have showed that SeNPs favor suppression of water deficit (Ikram et al., 2020), heat stress (Sita et al., 2022), and salt stress (Rasool et al., 2022). Overall, agricultural experiments should be conducted to determine the best dosages of selenium for various plant species as well as the proper timing of administration, particularly for those used to combat abiotic stressors. Additionally, the omics technique can quickly and accurately produce data that correlates a crop’s responses to its performance under various environmental situations.

### 2.4 GABA

GABA is a four-carbon non-protein amino acid that acts as a signaling and defense molecule in plant tissues and organs (Breitkreuz and Shelp, 1995; Wang et al., 2017; Balfagón et al., 2021). It comprises a significant fraction of the free amino acid pool in plant cells, serving as an important neurotransmitter while being involved in alleviating abiotic stresses (Nicolas Bouché, 2004; Nayyar et al., 2014; Baluška et al., 2020). The exogenous activity of GABA simulates the effects of stress on growth and development and increases endogenous GABA concentrations in tissues in response to diverse abiotic influences (Michaeli and Fromm, 2015). GABA accumulation in response to different abiotic and biotic stresses validates its role as a signaling molecule (Breitkreuz and Shelp, 1995; Ramesh et al., 2015). In *Arabidopsis* and *Brassica napus*, GABA is suggested to regulate nitrate uptake and improve nodule formation in *Medicago sativa* (Beuve et al., 2004; Barbosa et al., 2010). Furthermore, GABA participates in regulating leaf senescence and the plant circadian clock (Allan et al., 2008).

The effect of stress on crop expansion and growth is reduced through exogenous GABA control. Notably, GABA metabolite application in maize seedlings results in improved growth, reduced plant cell membrane injury, and enhanced soluble sugar and proline level. It also inhibits water loss under salt stress (Wang et al., 2017; Nephali et al., 2020; Kaspal et al., 2021). Abdel Razik et al. (2021) examined the effects of GABA on sunflower and found that it resulted in increase in chlorophyll and sugar concentrations via upregulation of antioxidant related defense enzymes under high temperature and drought stress (**Table 1**). The treatment of GABA against chilling injury on cold sensitive tomatoes resulted in enhancement of plant growth and development, as it upregulate cell expansion and antioxidant capacity (Liu et al., 2020). Other studies have demonstrated that GABA supplementation to wheat seedlings resulted in reduced ROS production, enhanced soluble protein biosynthesis, and regulated cellular amino acid balance (Sita and Kumar, 2020). While GABA application enhances reproductive function in mung beans under heat stress (Priya et al., 2019; Wang et al., 2021), it reduces ROS biosynthesis by upregulating osmolytes, resulting in sustained cell morphology and enhanced cellular functions under salt stress in various crops (Sita and Kumar, 2020). GABA mediated the activation of the Abscisic acid (ABA) signal pathway and improves the drought resistance of apple seedlings (Liu et al., 2021). ABA is a valuable plant hormone that controls plant response to environmental stresses and plays an important function in plant development and growth (Tuteja, 2007; Kaspal et al., 2021). Numerous abiotic stressors, such as salinity, heat, and drought, can result in increased ABA levels and regulated ethylene levels (Morgan and Drew, 1997; Kathiresan et al., 2018; Khalil et al., 2022). When GABA was applied, sunflower seedlings produced more ethylene (concentrations dependent; 0-300 mM). This was due to an increase in the expression of 1-aminocyclopropane-1-carboxylic acid synthesis (Kaspal et al., 2021).

The effect of GABA priming in creeping bent grass (*Agrostis capillaris*) on increasing plant resistance against abiotic stress was correlated with modifications in the levels of endogenous polyamines, amino acids, and sugars content under heat, drought, and salt stress (Li et al., 2020; Wang et al., 2020). Sita and Kumar (2020) linked abiotic stress tolerance in legumes with the metabolic and signaling functions of GABA and suggested that GABA can provide several health benefits. As pulses contain high amounts of GABA, developing legume-based functional foods will favor populations that suffer from chronic diseases. Despite significant advancements in the study of GABA in agricultural plants. In recent years, further investigation is necessary to fully comprehend the molecular processes underlying the protective benefits of GABA in stress endurance and to develop practical agricultural applications.

### 2.5 Seaweed extracts

Vast differences in seaweed extract composition produced using various extraction techniques and unique ingredients, may significantly affect plant response. Seaweed exhibits dual functionality as both a plant biostimulant and soil enhancer, promoting plant growth under stress conditions such as chilling, water deficit, and salt stress; it enhances photosynthetic activity, thereby improving the yield of various crops (Nephali et al., 2020; Banakar et al., 2022). Seaweed extracts are rich in carbohydrates, enzymes and proteins, and can be used to reduce abiotic stress, increase nutrient utilization, and stimulate root growth, quality, weight and microbial activity in the root zone of orange and other plants (Battacharyya et al., 2015; Sible et al., 2021; Lau et al., 2022). The effects of seaweed biostimulant have been linked with the plant growth hormones such as cytokinins and other low molecular weight compounds found in seaweed extracts (Safaei et al., 2022). The application of seaweed extracts resulted in improved growth and functioning of grapevines (*Vitis vinifera*) by enhancing resistance to water deficit stress (Monteiro et al., 2022; Samuels et al., 2022). Moreover, seaweed extracts can effectively improve plant nutrient assimilation and fruit development; enhance stress alleviation due to water deficit, salinity, drought, and heat conditions and improve yield in several horticultural and arable crops by increasing antioxidant enzyme activity and decreasing MDA levels (Mukherjee and Patel, 2020; Jacomassi et al. (2022). Seaweed extracts helped to promote the development and productivity of cowpea and maize (*Vigna sinensis* and *Zea mays*) plants. under salt stress (Hussein et al., 2021; Sleighter et al., 2023). Hernández-Herrera et al. (2014) revealed that most economically available seaweed extracts were obtained from brown seaweeds, including *Ascophyllum*, *Fucus*, and *Laminaria*. The application of Algafect (extracts from *Ascophyllum nodosum*, *Fucus* spp., and *Laminaria* spp.) at 16 mg kg^−1^ decreased leaf damage, increased shoot and root growth, and promoted root length and density in maize plants (Bradáčová et al., 2016).

Seaweed extract can stimulate the expression of genes encoding transporters of micronutrients (e.g., Cu, Fe and Zn) in *B. napus*, while improving the mineral composition of plant tissues (Billard et al., 2014; Kapoore et al., 2021). A seaweed extract-based biostimulant (*Dunaliella salina*) enriched in sulfated exopolysaccharides was found to increase proline, phenolic, and osmo-protectant chemical substance levels and enzyme activities, thereby alleviating salt stress in tomato plants (**Table 1**; El Arroussi et al., 2018). Notably, seaweed extracts can enhance nutrient uptake and improve growth performance in crops under stressed and normal conditions, as they contain several active compounds such as polysaccharides, polyphenols and phytohormones (Deolu-Ajayi et al., 2022). Initially, the beneficial effects observed in plants from seaweed products were attributed to the presence of multiple mineral elements in soluble forms. These elements were believed to play crucial roles during the vegetative phase in olive plants (Graziani et al., 2022). The presence of many bioactive molecules in seaweed biomass was subsequently found to directly affect plant physiology and metabolism while regulating the production and accumulation of endogenous metabolites involved in these biological processes (Bhattacharyya and Pal, 2015) The variability of the seaweed extract effect on plants makes it challenging for the research community to identify and separate the active ingredients in these products. Therefore, a need for standardization is arises in order to recognize and describe how these seaweed extracts impact plants.

### 2.6 Humic acids

Humic acid (HA) is a key component of humic substances (HSs) which are widely found in the environment. These materials undergo humification through organic matter, primarily derived from plants (Cha et al., 2020; Picchi et al., 2021). HAs are considered key priming agents to increase the production of some key plant biochemicals such as nucleic acids, vitamins, amino acids, and nutrients; they can improve the physicochemical properties of soil (Sangeetha et al., 2006; Kaya et al., 2020), by enhancing soil structure, cation exchange capacity, and nutrient and water retention thereby improving abiotic stress tolerance in plants (Baía et al., 2020).

Plants exhibit various responses to water deficit, encompassing physiological environmental conditions (Shehab et al., 2010). Plant sensitivity to drought varies depending on the species, level of stress, and development stage (Lewandrowski et al., 2017). Due to their physicochemical associations with soil particles, water, and metallic nutrients for plants, humic compounds can improve soil fertility (Cha et al., 2020). The mechanism of HAs in promoting plant growth may involve the enhancement of nutrient uptake and reduction of toxic element uptake (Russo and Berlyn, 1991; Masciandaro et al., 2002; Adil, 2012). Treatments of HAs and phosphorus were applied to pepper plants to alleviate abiotic stress (water deficit, salt and oxidative stress). The results showed improvements in crop health due to decreased Na content as well as enhanced macro/micro essential elements in roots and shoots (Çimrin et al., 2010; Van Oosten et al., 2017).

To actively regulate and decrease oxidative stress, plants utilize ROS scavenging mechanisms, including (i) an enzymatic antioxidant system and (ii) a non-enzymatic antioxidant mechanism commonly referred to as the “low molecular weight” antioxidant system (Tuteja, 2007; Choudhury et al., 2017). Salt stress also causes the accumulation of ROS, which results in oxidative-stress-induced toxic effects in plants (Hasanuzzaman et al., 2020; Khalil et al., 2022). HA application activated antioxidant related and ROS scavenging enzyme activities in rice (Schiavon et al., 2010). Such enzymes are essential for deactivating free O_2_ radicals synthesized in plants under water deficit and saline stress (Nephali et al., 2020). Cha et al. (2020) and Benito et al. (2022) reported that HAs enhanced high temperature stress tolerance in *Arabidopsis* plants by transcriptionally activating the heat-shock proteins. HAs can protect early seedlings of sorghum by enhancing plant tolerance under salt stress (Ali et al., 2020).

In common beans (*Phaseolus vulgaris* L.) treated with HAs at high salinity (120 mM NaCl), increased endogenous proline levels and decreased membrane diffusion signify adaptation to a higher saline environment (**Table 1**; Adil, 2012). Glycine betaine and proline can induce increased resistance to environmental conditions such as freezing, salt, drought, and oxidative stress in plants (du Jardin, 2015). Metabolomic analysis revealed that *Dunaliella salina* is enriched with sulfated exopolysaccharides, which upregulate levels of proline, phenolics, osmoprotectants, and antioxidant enzyme activities and reduce salt stress (García et al., 2012; Paul et al., 2019; Albadwawi et al., 2022). Jindo et al. (2020) demonstrated that HAs exerted multiple positive effects on crops: (i) enhancing soil structure, (ii) increasing phosphorus availability, (iii) balancing soil pH, (iv) promoting lateral root growth, and (v) stimulating nitrate absorption. With current investigations revealing the beneficial effects of biostimulants on plant health, conducting a meta-analysis of biostimulant effects on plant development could be a suitable approach. Nevertheless, the presence of numerous variables, such as different species, testing settings, and compositions, makes it challenging to obtain conclusive results.

### 2.7 Chitosan

Chitosan (Cs) is a biopolymer derived from the deacetylation of nontoxic and biofunctional chitin found in the exoskeleton of crustaceans (Zhao et al., 2019; Wang et al., 2021), and can be used to manage various biotic and abiotic stresses in plants (Hadwiger, 2013; Malerba and Cerana, 2020). Cs causes several biotic and abiotic stress-related defensive reactions in plants (Ahmed et al., 2022). The use of Cs as an elicitor can effectively address the difficulties in stress adaption resulting from abiotic and biotic stresses due to environmental challenges and increased food demand, which results in the unsustainable usage of synthetic chemicals (Hidangmayum et al., 2019; Malerba and Cerana, 2020).

Chitosan is a matrix for encapsulating and sequestering bioactive compounds (Malerba and Cerana, 2019). Chitosan–selenium NPs have been used as carriers for the slow release and adsorption of fertilizers, pesticides, herbicides, and plant growth regulators (Amer and Ibrahim, 2019). Sheikhalipour et al. (2021) reported that the use of foliar application of Cs-NPs can prevent potentially harmful effects on plants to improve plant yield (**Table 1**). Nephali et al. (2020) demonstrated the active role of Cs in mitigating the deteriorative impacts of salt stress on plant yield, growth, development and biomass by upregulating cellular ion translocation, managing osmotic balance and enhancing antioxidant-related enzymatic activities in lettuce. Hassan et al. (2021) reported that Cs-NPs efficiently counter salt stress by increasing antioxidant-related enzyme activity and alkaloid generation in periwinkle plant (*Catharanthus roseus*). The potential of Cs-NPs to increase the leaf antioxidant pool both in greenhouse and field plants was attributed to the slower release of chitosan due to nano‐formulation (Picchi et al., 2021). It induces the enzymatic antioxidant system and H_2_O_2_ scavenging, which increase membrane integrity and consequently improve plant tolerance to salt stress (Safikhan et al., 2018; Sen et al., 2020; Zulfiqar and Ashraf, 2021b).

Water scarcity, identified as the primary constraint to global food production (FAO, 2002), can be addressed using the topical application of chitosan. This approach has been shown to enhance water deficit resistance in maize hybrids (*Z. mays* L.) (Khordadi Veramin et al., 2019) and reduce plant damage caused by drought stress sesame (*Sesamum indicum*) (Veroneze-Júnior et al., 2020). Pretreatment of chitosan in various plants, prior to exposure to abiotic stresses, such as water deficit, salt, and heat stresses, was beneficial in achieving the desired plant growth and development owing to the enhanced activities of antioxidant-related enzymes (Pongprayoon et al., 2022).

Foliar application of chitosan resulted in enhanced crop tolerance against cadmium, ozone and oxidative stress in wheat (Liu et al., 2021; Picchi et al., 2021). Moreover, chitosan application stimulates germination and alters the expression of resistance genes in chili pepper, preventing the significant decrease in chili yield imposed by *Phytophthora capsici* (Esyanti et al., 2019). The most promising strategies for mitigating multiple stressors and enabling significant development in the productivity of plants are chitin and chitosan (Malerba and Cerana, 2019). Notably, both chitin and chitosan can be applied directly as natural fertilizers due to their substantial nitrogen levels and low C/N ratio (Shamshina et al., 2019). Despite positive findings, further effort in terms of production and research is required to ensure consistency in these elements and fully maximize the potential of chitosan significance.

### 2.8 Protein hydrolysate

Protein hydrolysates (PHs) are a category of plant biostimulants, including mixtures of polypeptides, oligopeptides and amino acids, which are manufactured from protein sources using partial hydrolysis (Schaafsma, 2009; Colla et al., 2014). Plant-derived PHs are increasingly well-known as plant biostimulants due to their capability to improve the emergence, production, and nutritional value of diverse horticultural and agronomic crops. The chemical hydrolysis of animal or microbial by-products comprise >90% of the PH (Colla et al., 2014); while enzymatic hydrolysis is typically used for producing plant-based protein hydrolysates (Lisiecka, 2011).

PHs are examples of plant- and animal-derived stimulants capable of increasing a plant’s tolerance to various abiotic stresses while enhancing plant growth and performance-related metrics like root growth/diameter, flowering, nutrient use efficiency/translocation, soil water holding capacity, and bacterial activity (Huang et al., 2011; Colla et al., 2014). PHs can stimulate N-metabolism and assimilation (Ertani et al., 2009; Baglieri et al., 2014). PHs and amino acids, including proline and betaine, favor the induction of secondary plant metabolism and increase plant defense responses and tolerance to various abiotic stresses, such as salinity, drought, temperature, heavy metals, and oxidative conditions (Apone et al., 2010; Ertani et al., 2013; Francesca et al., 2022). PHs can enhance nutrient availability in soils for plant development as well as the absorption of nutrients and application reliability in plants, resulting in indirect effects. The advantageous effects of PHs on plants could be attributed, in part, to the stimulation of plant microbiomes. Amino acids and amides serve as the primary organic nitrogen transport forms in nearly all plants. These compounds can be utilized directly for protein synthesis and the production of other necessary nitrogen compounds, or they can undergo metabolism (Rentsch et al., 2007). While PH can help promote plant growth, yield, and stress tolerance by enhancing amino acid absorption and transport. They can stimulate fine root growth and thereby improve the root capacity for nutrient uptake. Moreover, it can also enhance soil microbial and enzymatic activities to induce improved biological fertility (Lisiecka, 2011).

Several studies have shown PH-enhancing effects on plant growth, development, and yield. These beneficial effects may be attributed to the direct influence of bioactive compounds (signaling peptides and free amino acids) on plant metabolism and the indirect impact resulting from the stimulation of plant microbiomes (Ertani et al., 2009; Paul et al., 2019). Colla et al. (2014) and Francesca et al. (2022) showed the positive impacts of plant-derived PHs on the growth aspects of maize, pea, and tomato (**Table 1**). In another study, PH derived from alfalfa plants enhanced shoot biomass production, accumulation of soluble sugars and nitrogen assimilation in hydroponically grown maize plants (Nardi et al., 2016; Hamid et al., 2021). This biostimulant produced from alfalfa plants improves the short-term development of maize under salt stress by promoting the expression of phenylalanine ammonia-lyase enzymes and genes as well as flavonoid synthesis (Ertani et al., 2009; Ertani et al., 2013). Tomato plants treated with a PH-based biostimulant; rich in glycine betaine, glutamic acid, and micronutrients as manganese, boron, and zinc, had better water status and pollen viability and demonstrated higher yield and antioxidant level (Francesca et al., 2021).The above findings explain why the PH-treated plants usually exhibit enhanced nutrient uptake and assimilation. The usefulness of PH usage in tomato to improve plant performance was reported under limited water availability (Francesca et al., 2022). To enable crops to survive environmental challenges, PH are widely used in agriculture as plant biostimulants. They can exhibit biostimulatory function by upregulating some essential enzymes involved in carbon-nitrogen metabolism and increasing the function of antioxidant enzymes and the synthesis of secondary metabolites (Rouphael and Colla, 2018; Lau et al., 2022). PHs offer significant potential for tackling the dual challenges of feeding expanding populations mitigating the negative impacts of agriculture on the environment and human health. Further research is needed to understand the mechanisms underlying the beneficial effects of these substances and to develop optimal product formulations and management practices that maximize their positive impact across diverse agro-ecological conditions.

### 2.9 Microbial biostimulants

**Figure 2:**
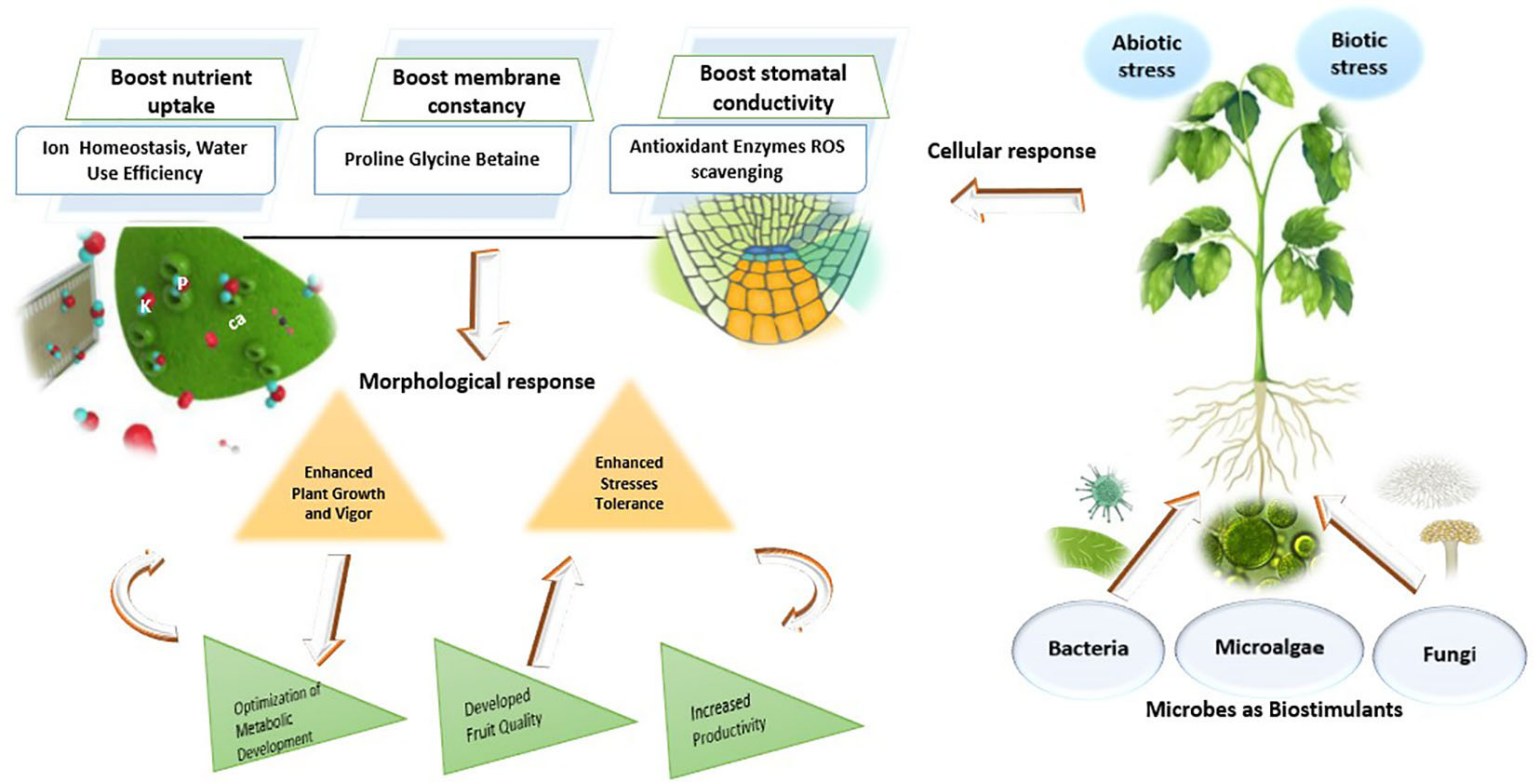
Mechanisms of microbial biostimulants influence on plant growth under stress conditions.

The use of bio-fertilizers and biostimulants, as sustainable alternatives, could potentially mitigate the effects of abiotic and biotic stresses and enhance the quality and production of crops. Environmentally friendly materials known as biofertilizers, which are applied in substantial amounts, include microbes e.g. bacteria, fungi, yeast or microalgae that enhance the growth and development of plants by inhabiting rhizospheres and increasing their ability to absorb nutrients such as nitrogen, phosphorus, potassium, and minerals (Ritika; and Utpal, 2014; Win et al., 2018; Bulgari et al., 2019; Kapoore et al., 2021) (**Figure 2**).

**Figure 3:**
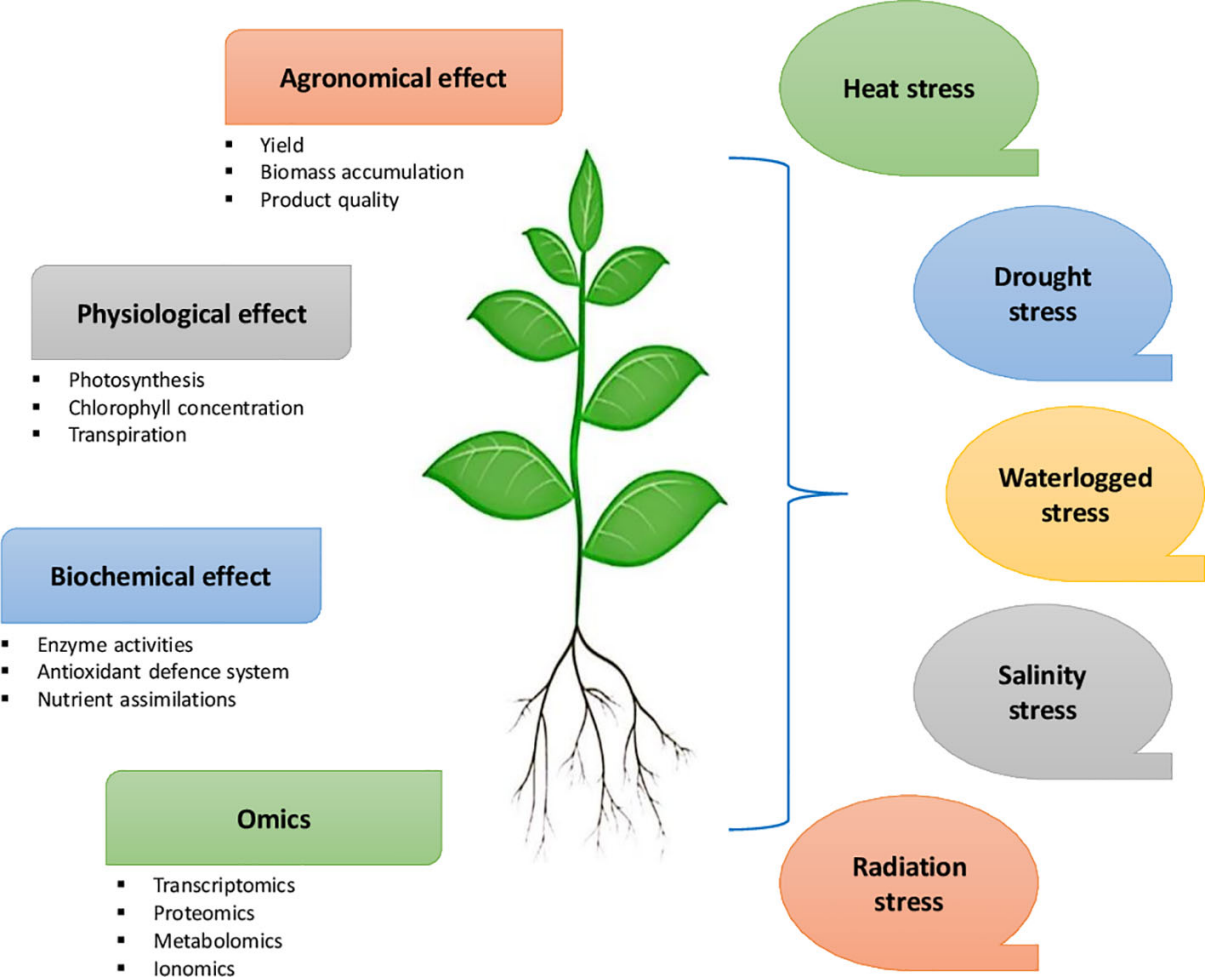
Effect of different abiotic stresses on agronomic, physiological, biochemical and molecular functions of plants that may occur at different developmental stages.

Microbial plant biostimulants (MPBs) such as *Arthrobacter*, *Azotobacter, Azospirillum, Bacillus, Pseudomonas*, and *arbuscular mycorrhizal* fungi (AMF) can enhance plant growth and mitigate abiotic stresses in crops such as tomato, potato, soybean, cabbage, broccoli, maize, and rice (Lubna et al., 2018; Kim et al., 2020; Kubi et al., 2021). While abiotic stresses induce physiological, biochemical, and molecular effects on plants (**Figure 3**), they have various implications on the soil microbial diversity, which influence key plant functional traits (Ali et al., 2022). MPBs support plant nutrition and induce significant changes in secondary metabolism and tolerance to soil and environmental stresses (Rouphael et al., 2020). Combining different biostimulants may have synergistic effects in combating biotic and abiotic stresses in crop plants. Furlan et al. (2019) found that the application of the biostimulant combination Nutrifer202 (includes in its composition an algal extract) and *Bradyrhizobium* sp. C-145 was promising for peanut crops growing in regions susceptible to water deficit or arsenic exposure. The application reduced arsenic translocation to leaves and improved plant growth and root nodulation, in association with proline accumulation (**Table 2**). Ruzzi and Ricardo (2015) suggested using to inoculate plant growth-promoting rhizobacteria bacteria (PGPR) for the desired effects in plants. The application of living cyanobacteria is recognized as a potential biocontrol substance that stimulates the production of defense enzymes and provides hydrolytic enzymes and antibacterial chemicals to combat plant diseases (Dasgan et al., 2012; Gupta et al., 2013; Garcia-Gonzalez and Sommerfeld, 2016). Furthermore, Koleška et al. (2017) reported that biostimulants prevented yield loss and reduced oxidative damage in tomato plants grown on reduced NPK nutrition. Synergizing biostimulants with reduced NPK fertilizer stabilized of cell homeostasis in tomato plants with enhanced adaptation to stress conditions

**Table 2:** Examples of commercial PGPR-based products.

	Crop	Products	Compositions
Cereal crops		Nitroguard®	*Azoarcus indigens NAB04, Azorhizobium. caulinodens NAB38, Azospirillum brasilense NAB317, Bacillus* sp.
		Micosat F® Cereali	*Streptomyces* spp. *ST 60, Paenibacillus durus PD 76, Bacillus. subtilis BR 62.*
		BactoFil A10®	*Azotobacter vinelandii, A. brasilense, Pseudomonas fluorescens, B. megaterium, B. polymyxa*,
		Inomix® Biostimulant	*B. subtilis (IAB/BS/F1), B. polymyxa (IAB/BP/01.*,
		Inomix® Biofertilisant	*Saccharomyces cerevisiae, B. megaterium, Rhizobium leguminosarum, A. vinelandii*,
		Inomix® phosphore Rhizocell ® GC	*P. fluorescens, B. amyloliquefaciens souche IT45, B. megaterium, S. cerevisiae*
Horticultural crops	Fruits, vegetables, ornamental	Ceres®	*Pseudomonas fluorescens*
		FZB24®fl; Rhizovital 42®	*B. amyloliquefaciens ssp. Plantarum bamyloliquefaciens*
		Gmax® PGPR	*Azotobacter, Phosphobacteria, P. fluoresces*
		Micosat F® Uno	*A. radiobacter AR 39, B. subtilis BA 41, Streptomyces* spp. *SB 14*
		Amase®	*Pseudomonas azotoformans*
	Vegetables (Cucumber, lettuce, tomato, pepper)	AmniteA100®	*Azotobacter, Bacillus, Pseudomonas, Rhizobium, Chaetomium*
		Micosat F® Cereali	*B. subtilis BR 62, P. durus PD 76, Streptomyces* spp. *ST 60*
		Symbion®-N	*Azospirillum, Rhizobium, Acetobacter, Azotobacter*
		TwinN®	*A. brasilense NAB317, A. caulinodens NAB38, A. indigens NAB04*
		Symbion®-P	*B. megaterium* var. *phosphaticum*
		Symbion®-K	*Frateuria aurantia*
Oil Crops	Sunflower, Oilseed Rape	BactoFil B10®	*Azospirillum lipoferum, A. vinelandii, B. megaterium, B. circulans*, *B. subtilis, P. luorescens*
	Sunflowers, Soybeans	Micosat F® Cereali	*B. subtilis BR 62, P. durus PD 76, Streptomyces* spp. *ST 60*
	Oilseed Rape	Nitroguard®	*A. brasilense NAB317, Azorhizobium caulinodens NAB38, A. indigens NAB04, Bacillus* sp.
Other crops	Sugar beet, Sugarcane,	TwinN®; Nitroguard®	*A. brasilense NAB317, A. caulinodens NAB38, A. indigens NAB04*
	Potato	BactoFil B10®	*A. lipoferum, A.* *vinelandii, B. megaterium, B. circulans*, *B. subtilis, P. fluorescens*

Microalgae are a varied group of primarily single-celled organisms that utilize photosynthesis to convert light and CO_2_ into various compounds. They are ecologically sound alternatives for improving and sustaining plants as they can enhance soil conditions by regenerating interactions between bacteria (Garcia-Gonzalez and Sommerfeld, 2016; Kapoore et al., 2021). To date, microalgae have been intensively investigated for their potential applications in biofuels, aquaculture, animal feeds, bioremediation, nutraceuticals, medicines, and cosmeceuticals (Chanda et al., 2019). Salt stress on crops is diminished by treatment with microalgae. Ion osmotic stress is caused by excessive salt, which is hazardous to plants (Zhu, 2001). Different application rates of microalga extract have been used to increase plant vegetative growth parameters, leaf chlorophyll level, nutrient use efficacy, and total protein percentage (Kumar et al., 2022). In wheat, microalgae significantly reduced the production of superoxide radicals, increased the abundance of antioxidant enzymes, and consequently improved the salt tolerance of wheat, implying that salt stress alters oxidative metabolism (El-Baky et al., 2010; Kapoore et al., 2021). Spirulina microalgae acts as biostimulants in snap bean cv. ‘Valentine’ during plant growth, resulting in a 6% increase in normal growth yields and up to a 10% yield when combined with biostimulants. It also enhances plant growth parameters including leaf area per plant, and dry weight (Kumar et al., 2022). The use of algae extract-based biostimulants incorporating zinc and manganese in maize crops promotes plant cold resistance/tolerance by improving the elimination of ROS mechanisms (Bradáčová et al., 2016; Kaya et al., 2020).

Tarakhovskaya et al. (2007) revealed that micro- and macro-algae comprise phytohormones such as auxins, cytokinins, GAs, and brassinosteroids (BRs). The biosynthesis and signaling mechanisms of phytohormones in microalgae were recently detected through genomic research (Kenrick and Crane, 1997; Kapoore et al., 2021). Microalgae, by maintaining phytohormones in their cells and releasing them into the extracellular environment, offer various biostimulants capable of enhancing the resilience and sustainability of agriculture.

Despite these beneficial outcomes, microbial biostimulants may exhibit variable effects across different agricultural products or locations. To enhance the effectiveness and consistency of microbial biostimulants, further research is required to explore specific microbes with targeted functions. This includes utilizing species-specific microbes for soil restoration, improving nutrient uptake limited conditions, and saline regions to increase plant resilience against drought and salt stress (Khalil et al., 2022). The study of microbe’s applications could be beneficial in preparing for potential challenges in agriculture based on the aforementioned methods.

## 3 Conclusion

Intensive agricultural research must focus on promoting sustainable agricultural ecosystems, conserving water resources, improving soil health, and increasing plant tolerance to abiotic stresses. These fundamental concepts are crucial for ensuring the safety and quality of agricultural products and mitigating irreversible losses. Biostimulants are essential for reducing reliance on synthetic chemicals and improving plant physiology and metabolism under abiotic stress through various mechanisms, such as the regulation of phytohormones, signalling pathways and gene regulation; enhancement of bioactive compounds, and optimization of ROS enzyme activities. Different biostimulants including seaweed extracts, zinc oxide, silicon, humic substances, chitosan, exudates, and other microbial and nonmicrobial biostimulants, can counter abiotic stress while ensuring ecological and human health safety. Despite the confusion in classifying certain agents as biostimulants, particularly hormones that play significant role beyond traditional plant growth substances. Expanding the use of these biostimulants to several crops and types for commercialization in order to ensure food and nutritional security is necessary. Furthermore, investigating the potential positive effects of plant biostimulants, specifically those based on PGPR, in the context of climate change will be worthwhile. Agriculture sector accounts for approximately 21% of the global greenhouse effect, with 13% resulting from chemical fertilizer utilization. Research and experimentation in this regard must rapidly provide valuable insights to improve biostimulant production and to optimize their techniques of application. Notably, numerous biostimulant treatments can be used in future agriculture research to enhance commercial yield under various stresses, as this will contribute to increased annual global food productivity while minimizing costs and reducing negative impacts on the environment. Despite the potential benefits of microbial biostimulants, their effectiveness and environmental sensitivity are still considered drawbacks when compared to nonmicrobial biostimulants. The utilization of microbial biostimulants can augment human contributions to agricultural ecosystems by offering a sustainable and effective solution to mitigate production losses caused by climate change. The development of future generations of biostimulant products holds promise for exploring synergistic effects when combining multiple types of biostimulants. Maximizing the beneficial impact through the development of multispecies formulations is significantly important. Overall, biostimulants offer the potential for improving soilless cultivation techniques.

## Author contributions

ZA: Conceptualization, Data curation, Formal analysis, Funding acquisition, Investigation, Methodology, Project administration, Supervision, Visualization, Writing – review & editing. AA: Investigation, Methodology, Writing – original draft. MA: Investigation, Methodology, Writing – original draft. MQ: Data curation, Project administration, Software, Writing – original draft. RKa: Supervision, Visualization, Writing – review & editing. ZS: Formal analysis, Validation, Writing – review & editing. RKh: Data curation, Validation, Visualization, Writing – review & editing. FG: Formal analysis, Visualization, Writing – review & editing.
